# Prevalence, associated factors, and machine learning–based prediction of infertility among married women in North Shewa Zone, Ethiopia: a cross-sectional study

**DOI:** 10.1186/s12905-026-04724-4

**Published:** 2026-07-28

**Authors:** Abate Tadesse Zeleke, Tadesse Ayele Belachew, Merga Abdissa Aga, Degemu Sahlu Asebe, Rebik Shukure Beyane

**Affiliations:** 1 Department of Statistics, Salale University, Fiche, Ethiopia; 2 Department of Public Health, Salale University, Fiche, Ethiopia

**Keywords:** Infertility, Machine learning, Random forest, Logistic regression, Reproductive health, Ethiopia

## Abstract

**Background:**

Infertility, defined as the inability to conceive after 12 months of regular unprotected sexual intercourse, is an important public health concern with significant social, psychological, and marital consequences. In this study, infertility was based on self-reported time to conception rather than clinical diagnosis; no clinical examination or male factor assessment was conducted, and outcome misclassification is therefore possible. Despite its importance, community-based evidence in Ethiopia remains limited. This study aimed to estimate the prevalence of infertility, identify factors associated with infertility, and evaluate the predictive performance of selected machine learning models among married women in North Shewa Zone, Oromia, Ethiopia.

**Methods:**

A community-based cross-sectional study was conducted among 829 randomly selected married women from three districts. Infertility status was self-reported based on time to conception. Multivariable logistic regression with Firth penalization was used to identify factors associated with infertility, and adjusted odds ratios (AORs) with 95% confidence intervals (CIs) were reported. For predictive analysis, machine learning models—including Random Forest (RF), Extreme Gradient Boosting (XGBoost), and Support Vector Machine (SVM)—were developed using an 80/20 train–test split with 5-fold cross-validation applied to the training data. Class imbalance was addressed using resampling techniques. Model performance was evaluated using accuracy, sensitivity, specificity, and area under the receiver operating characteristic curve (AUC).

**Results:**

The prevalence of infertility was 12.7%. Factors significantly associated with infertility included district of residence, age at marriage, irregular menstrual cycles, history of abortion, family history of infertility, number of sexual partners, cigarette smoking, and women’s occupation. In predictive modelling, Random Forest achieved the highest AUC (0.89), followed by XGBoost (0.88) and SVM (0.81), compared with logistic regression (AUC = 0.69). However, differences in predictive performance were modest. Given the relatively small number of infertility events, there is potential for model instability and overfitting. Variable importance analysis from the Random Forest model identified women’s occupation, education level, family history of infertility, and menstrual cycle pattern as important predictors.

**Conclusion:**

Infertility among married women in North Shewa Zone is associated with multiple socio-demographic, reproductive, and behavioral factors. However, findings should be interpreted cautiously due to the cross-sectional design, self-reported outcome, absence of clinical verification, and limited number of events. Although machine learning models demonstrated relatively higher predictive performance, results are based on internal validation only and may not generalize to other populations. Logistic regression remains appropriate for inference, while machine learning approaches may be useful for exploratory prediction. Further studies with larger sample sizes, improved outcome measurement, and external validation are required before practical application.

## Background

Infertility is defined by the World Health Organization (WHO) as the failure to achieve pregnancy after at least 12 months of regular, unprotected sexual intercourse among couples of reproductive age who are actively attempting conception [1, 2]. It is broadly classified into primary infertility, referring to the absence of any prior conception, and secondary infertility, which denotes failure to conceive following a previous pregnancy [3]. Infertility may result from female, male, or combined factors; however, in many settings, the inability to conceive is disproportionately attributed to women, leading to significant social and health consequences [3].

Globally, infertility affects millions of couples and remains a substantial public health concern across both high- and low-income countries [2]. Beyond its biological implications, infertility is associated with psychological distress, social stigma, marital instability, and economic hardship. Evidence from regions such as South Asia and the Middle East indicates that infertility may also increase women’s vulnerability to interpersonal violence, highlighting its broader social implications [4, 5].

In Ethiopia, infertility remains an important reproductive health issue. Cultural norms place a strong emphasis on childbearing, often assigning women primary responsibility for reproduction regardless of the underlying cause. Consequently, women frequently experience stigma, marital instability, and psychological distress in the context of infertility [3, 6, 7].

Available evidence in Ethiopia is limited and largely derived from facility-based studies, which may not accurately represent the general population. For instance, a study conducted in Addis Ababa reported an infertility prevalence of 27.6% among women attending health facilities [6]. Qualitative findings have also suggested that factors such as advanced maternal age (commonly defined as ≥ 35 years), anxiety, contraceptive use, and reproductive health history may influence infertility experiences [7]. Another study from Dessie identified factors including age at first pregnancy, age at menarche, menstrual characteristics, history of sexually transmitted infections, and multiple sexual partners as associated with infertility [3]. However, these findings are primarily based on clinical populations and may not be generalizable to community settings.

Although previous studies have identified demographic, socioeconomic, reproductive, and behavioral correlates of infertility [3, 6–12], most have relied on conventional statistical approaches and have limited geographic coverage. Community-based evidence remains scarce, particularly in rural and semi-urban settings. Furthermore, the application of machine learning methods in infertility research in Ethiopia is still limited, despite their potential to improve predictive accuracy by capturing complex and non-linear relationships among variables.

Therefore, this study aimed to estimate the prevalence of infertility and identify associated factors among married women in North Shewa Zone, Oromia, Ethiopia, using a community-based cross-sectional design. In addition, the study evaluated the performance of selected machine learning models in predicting infertility and compared their performance with conventional logistic regression analysis.

## Methods

### Study setting

The study was conducted in North Shewa Zone, Oromia National Regional State, Ethiopia, from March to June 2023. The zone is located approximately 113 km north of Addis Ababa, with an altitude ranging from 2,738 to 2,782 m above sea level. Administratively, the zone comprises 18 districts with an estimated total population of 2,054,435, of whom a substantial proportion are women of reproductive age (15–49 years) [13].

### Study design and population

A community-based cross-sectional study design was employed, supplemented by a qualitative component using focus group discussions (FGDs). The study population consisted of married women aged 15–49 years who had lived with their spouse for at least one year in the selected districts (Dera, Girar Jarso, and Wachale).

Women were excluded if they were unmarried, unable to communicate due to hearing or speech impairment, or had severe mental illness at the time of data collection. Given the cross-sectional design, the analysis focuses on associations rather than causal relationships.

### Data collection procedures

Quantitative data were collected using a semi-structured interviewer-administered questionnaire adapted from previous studies [3, 6, 7, 10, 14–16]. The questionnaire primarily consisted of closed-ended questions to ensure consistency and facilitate quantitative analysis, while a limited number of semi-structured items were included to capture additional contextual information.

The questionnaire was initially prepared in English, translated into Afan Oromo and Amharic, and back-translated to ensure consistency. A pretest was conducted prior to the main survey. Data were collected by trained enumerators under close supervision.

For the qualitative component, two focus group discussions (FGDs) were conducted in each district. Participants were purposively selected to capture diverse perspectives. Discussions were guided by semi-structured questions focusing on perceptions, experiences, and social implications of infertility. Each FGD was moderated by a trained facilitator, audio-recorded with consent, and later transcribed and translated into English for analysis.

### Sampling technique and sample size determination

A three-stage probability sampling technique was used [17]. First, three districts (Dera, Girar Jarso, and Wachale) were selected using simple random sampling. Second, four kebeles were randomly selected from each district. Third, eligible married women were selected systematically from households within each selected kebele.

The sample size was determined using the single population proportion formula, assuming a 50% prevalence to yield the maximum sample size in the absence of prior population-based estimates. A 95% confidence level, 5% margin of error, and design effect of 2 were applied to account for the multistage sampling design [18]. The initial sample size for a simple random sample was calculated as:

$$\:{n}_{o}=\frac{\:{\:\:(\mathrm{Z}\:{\upalpha\:}/2\:)}^{2}\mathrm{p}\mathrm{q}}{{\mathrm{d}}^{2}}$$

$$n_o=\frac{\left(1.96\right)^20.5\ast0.5}{0.5^2}=384.16\;=\;385$$

The final sample size accounting for clustering was then calculated as:

$$n\;=\widetilde n\;\ast design\;effect=385\ast2=770$$

To compensate for potential non-response, 10% was added, resulting in a final sample size of 847 married women aged 15–49 years.

The total sample was allocated to each district using probability proportional to size (PPS), as presented in Table 1. In addition, two focus group discussions (FGDs) were conducted in each district to complement the quantitative findings.

**Table 1:** Proportional allocation of the study sample across selected districts

Sampled district	Target women population size	Sampled women per district
Dera	82,077	448
Girar Jarso	28,997	159
Wachale	43,928	240

Sampled Women 847

### Study variables

#### Response variables

Infertility was defined as self-reported infertility, operationalized as failure to achieve pregnancy after at least 12 months of regular unprotected sexual intercourse among women actively attempting conception, consistent with WHO criteria [2]. The outcome was coded as 1 = infertile and 0 = fertile.

However, infertility status was based solely on self-report and was not clinically verified, and no clinical examination or male factor assessment was performed, which may introduce outcome misclassification bias. Both primary and secondary infertility were combined into a single outcome variable.

Women who were pregnant, using contraception, or within the postpartum amenorrhea period were not considered at risk of conception and were excluded from classification.

#### Explanatory variables

Socio-demographic, socioeconomic, behavioral, reproductive, and biological factors were considered. Cigarette smoking was defined as current or past use, and number of sexual partners referred to lifetime sexual partners.

### Data management and conventional statistical analysis

#### Quantitative data analysis

Data were entered, cleaned, and analyzed using R software. Descriptive statistics were summarized using frequencies, percentages, tables, and graphs. Sampling weights were not applied in the analysis; therefore, the estimates may not be fully representative of the target population, and the findings should be interpreted with caution.

Bivariable logistic regression was conducted to assess crude associations between infertility and each explanatory variable. Variables with p-value < 0.25 in bivariable analysis, along with variables of established epidemiological relevance, were included in the multivariable logistic regression model. This threshold was used as a screening criterion to avoid excluding potentially important variables.

Adjusted odds ratios (AORs) with 95% confidence intervals (CIs) were reported. Statistical significance was declared at *p* < 0.05 [19].

Model assumptions were assessed prior to interpretation. Multicollinearity was evaluated using the Variance Inflation Factor (VIF), with VIF > 10 indicating potential concern. Model fit was assessed using the Hosmer–Lemeshow goodness-of-fit test, and discrimination ability was evaluated using the area under the ROC curve (AUC) [20, 21].

Given the multistage sampling design, clustering at the kebele level may affect standard errors. However, due to the absence of cluster identifiers in the final analytic dataset and relatively large within-cluster sample sizes, standard logistic regression was applied. This may result in underestimation of standard errors, and findings should therefore be interpreted cautiously.

Given the relatively small number of infertility cases relative to the number of predictors and sparse observations in some covariate categories, there was potential for sparse data bias and unstable estimates in the conventional logistic regression model. To address this, Firth’s penalized logistic regression was applied. This approach reduces small-sample bias and is particularly useful in the presence of rare events or separation. The results from the penalized model were compared with those from the standard logistic regression, and consistent findings were observed, with slightly attenuated effect estimates.

#### Qualitative data analysis

Qualitative data obtained from focus group discussions (FGDs) were analyzed using thematic analysis. Audio recordings were transcribed verbatim and translated into English. An initial coding framework was developed based on the interview guide and emerging concepts from the data. Transcripts were independently reviewed and coded by the research team, and codes were iteratively refined and grouped into broader themes.

To enhance the trustworthiness of the findings, multiple strategies were employed, including analyst triangulation, where more than one researcher participated in coding and interpretation, and consistency checks across transcripts. Discrepancies were discussed and resolved through consensus.

The final themes captured key perceptions, social experiences, and behavioral factors related to infertility. These qualitative findings were used to complement and contextualize the quantitative results, providing a more comprehensive understanding of infertility in the study setting.

### Machine learning analysis

#### Data preprocessing

Predictor variables were selected based on epidemiological relevance and data completeness [22]. Categorical variables were encoded using appropriate methods prior to model fitting [23]. Missing data were assessed prior to analysis. The proportion of missing values was low (< 5%) across all variables; therefore, a complete-case analysis was conducted. Given the low level of missing-ness, the impact on estimates is expected to be minimal. However, if missing-ness is not completely at random, some bias may remain.

The dataset was randomly split into training (80%) and testing (20%) subsets. All preprocessing steps, including encoding, handling of class imbalance, and model tuning, were conducted exclusively on the training set to prevent information leakage and ensure valid model evaluation [24].

#### Handling class imbalance

Given the relatively low prevalence of infertility, class imbalance was addressed using the Random Over-Sampling Examples (ROSE) technique applied to the training dataset [25].

ROSE generates synthetic samples using a smoothed bootstrap approach and may improve model sensitivity. However, it can introduce artificial patterns in small datasets. Therefore, model performance was also evaluated using the original (non-resampled) data to assess robustness.

Alternative approaches such as SMOTE, Borderline-SMOTE, ADASYN, and class weighting were considered but not implemented. These methods may offer advantages in certain contexts, and the choice of ROSE should not be interpreted as optimal.

### Machine learning models

Four models were implemented and compared: logistic regression (baseline), Random Forest (RF), Extreme Gradient Boosting (XGBoost), and Support Vector Machine (SVM) with a radial basis function kernel [26–28]. Logistic regression was included as a baseline inferential model for comparison rather than as a machine learning algorithm, given its primary use for estimating associations. The dataset was split into training (80%) and testing (20%) subsets.

Hyperparameter tuning was performed using grid search with 5-fold cross-validation on the training dataset. For Random Forest, the number of variables randomly selected at each split (mtry) and the number of trees were optimized. For XGBoost, key parameters including learning rate, maximum depth, and number of estimators were tuned. For SVM, the cost (C) and gamma parameters were optimized.

Hyperparameter tuning was conducted using grid search combined with 5-fold cross-validation on the training dataset. The predefined search space was as follows:

- Random Forest: mtry (√p, p/3), number of trees (100, 200, 500)
- XGBoost: learning rate (0.001, 0.01, 0.1), maximum depth (3, 5, 10), number of estimators (50, 100, 200)
- SVM: cost (0.01, 0.1, 1, 10), gamma (0.001, 0.01, 0.1)

Model performance during tuning was evaluated using accuracy and F1-score, and the optimal hyper-parameter combination was selected based on the highest mean cross-validation performance. Hyper-parameter tuning was strictly performed within the training folds to prevent information leakage.

The final selected hyper-parameters were:

- ➢ Random Forest: mtry = 3, number of trees = 500
- ➢ XGBoost: learning rate = 0.1, maximum depth = 5, number of estimators = 200
- ➢ SVM: cost (C) = 1, gamma = 0.01

Using these optimal parameters, the final models were retrained on the full training dataset and subsequently evaluated on the independent test set.

However, nested cross-validation was not implemented, and therefore hyperparameter tuning and model evaluation were not fully separated. This may result in optimistic bias in performance estimates.

In addition, given the relatively small number of infertility events (*n* = 105), the events-per-variable ratio is limited, which increases the risk of model instability and overfitting, particularly for more complex models such as Random Forest and XGBoost. As a result, model performance should be interpreted cautiously.

All analyses were conducted using R software (version 4.2.1). Machine learning models and tuning procedures were implemented using established R packages, including *caret*, *randomForest*, *xgboost*, and *e1071*.

### Model performance evaluation

Model performance was evaluated using accuracy, sensitivity, specificity, and the area under the receiver operating characteristic curve (AUC). Performance during training was assessed using 5-fold cross-validation, and results were summarized as mean ± standard deviation. Final model evaluation was conducted on an independent test dataset to assess generalization performance [29, 30].

Differences in AUC between models were assessed; however, formal statistical comparison using DeLong’s test was not consistently applied, and therefore observed differences should be interpreted with caution.

To address class imbalance, the Random Over-Sampling Examples (ROSE) technique was applied during model training. ROSE generates synthetic samples using a smoothed bootstrap approach and may reduce overfitting compared to simple random oversampling, particularly in small datasets with limited minority class observations. However, ROSE can introduce artificial patterns, and its use should not be considered optimal. Alternative approaches such as SMOTE, Borderline-SMOTE, ADASYN, and cost-sensitive learning (class weighting) were considered but not implemented. The absence of comparison with these methods represents a limitation.

Model calibration was assessed using the Brier score and calibration plots. However, additional calibration metrics, including calibration intercept and calibration slope, were not evaluated, which may limit the assessment of model reliability.

All machine learning results are based on internal validation only, and no external validation was performed, limiting generalizability and clinical applicability. Furthermore, the absence of nested cross-validation may result in optimistic performance estimates, as hyperparameter tuning and model evaluations were not fully separated.

#### Reporting guidelines

This study was conducted and reported in accordance with the STROBE (Strengthening the Reporting of Observational Studies in Epidemiology) guidelines, and the prediction modeling component was guided by the TRIPOD (Transparent Reporting of a Multivariable Prediction Model for Individual Prognosis or Diagnosis) statement.

## Results

### Prevalence of infertility

A total of 829 married women participated in the study, yielding a response rate of 97.9%. All respondents had been living with their partners for at least 12 months prior to the survey, and eligibility was restricted to women who reported attempting conception. The overall prevalence of infertility among married women aged 15–49 years was 12.7% (*n* = 105).

**Fig. 1:**
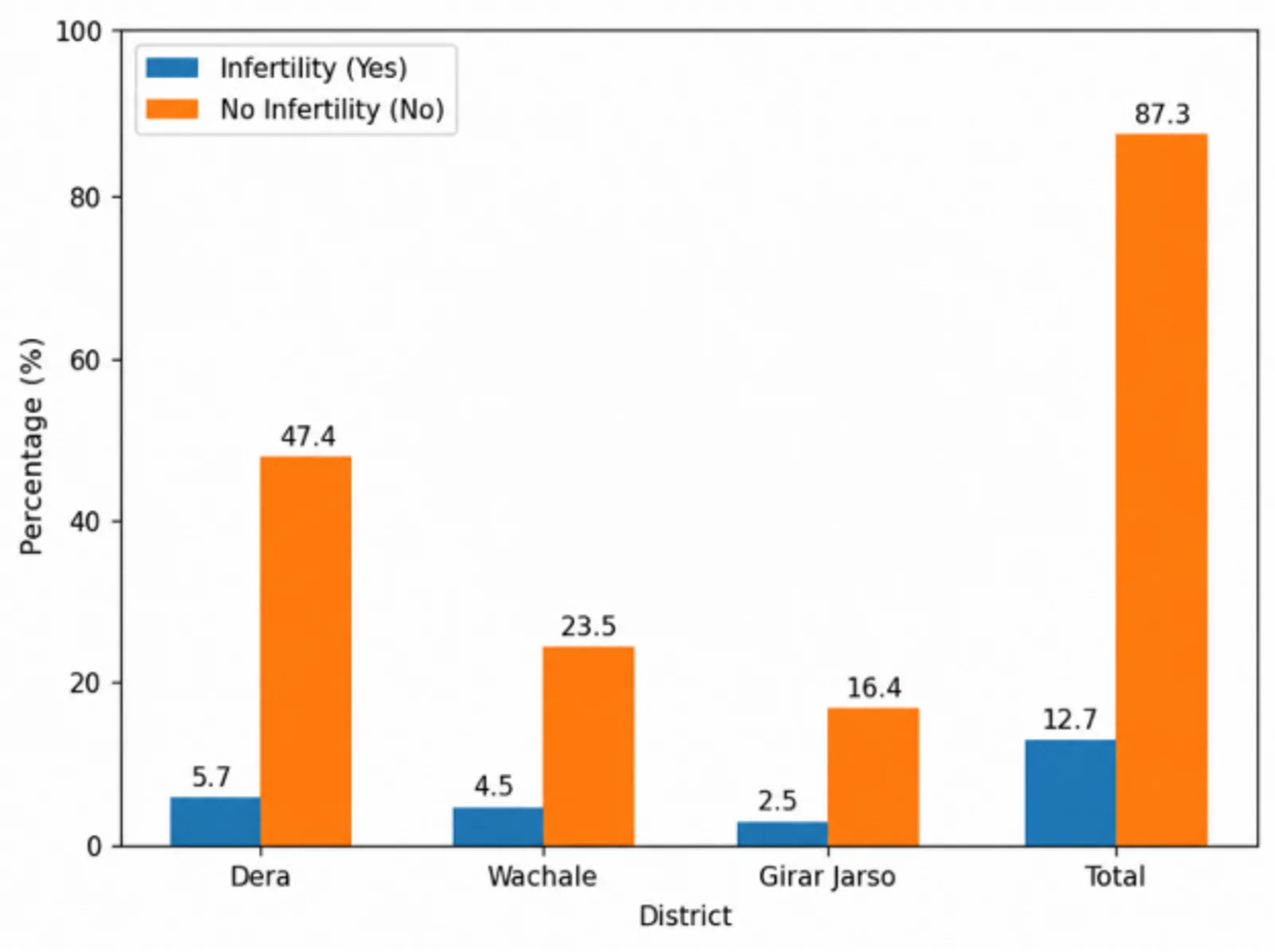
Distribution of infertility cases across districts as a proportion of the total sample

Among the total sample, 5.67%, 4.46%, and 2.53% of infertility cases were observed in Dera, Wachale, and Girar Jarso, respectively. Fig. 1 presents the distribution of infertility cases relative to the total sample, rather than district-specific prevalence. The higher proportion observed in Dera reflects its larger sample size and does not imply a higher underlying prevalence.

### Bivariable analysis: infertility and selected characteristics

Table 2 presents the results of the bivariable logistic regression analysis. Crude odds ratios (CORs) with 95% confidence intervals (CIs) were estimated to assess associations between infertility and selected characteristics.

**Table 2:** Bi-variable logistic regression analysis of factors associated with infertility

Variables	Categories	No Infertility *n* (%)	Infertility *n* (%)	COR (95% CI)	*p*-value
District (Ref: Dera)					
	G/Jarso	136 (86.6)	21 (13.4)	1.29(0.73–2.29)	0.379
	Wachale	195 (84.1)	37 (15.9)	1.58(0.96–2.60)	0.071
	Dera	393 (89.3)	47 (10.7)	1.00	—
Education (Ref: No education)					
	Primary	195 (83.7)	38 (16.3)	1.83(1.08–3.09)	0.024
	Secondary	147 (90.7)	15 (9.3)	0.96(0.49–1.89)	0.910
	Diploma+	119 (83.2)	24 (16.8)	1.89(1.01–3.54)	0.045
	No education	263 (90.4)	28 (9.6)	1.00	—
Residence (Ref: Urban)					
	Rural	280 (85.9)	46 (14.1)	1.28(0.83–1.98)	0.266
	Urban	444 (88.3)	59 (11.7)	1.00	—
Occupation (Ref: Housewife)					
	Farmer	85 (89.5)	10 (10.5)	1.33(0.63–2.81)	0.454
	Merchant	204 (81.0)	48 (19.0)	2.65(1.59–4.41)	< 0.001
	Government	130 (86.7)	20 (13.3)	1.74(0.94–3.23)	0.078
	Housewife	305 (91.9)	27 (8.1)	1.00	—
HH Head (Ref: Male)					
	Female	127 (77.9)	36 (22.1)	2.37(1.50–3.74)	< 0.001
	Male	577 (89.3)	69 (10.7)	1.00	—
Menstrual Cycle (Ref: Irregular)					
	Regular	406 (89.2)	49 (10.8)	0.68(0.45–1.02)	0.064
	Irregular	318 (85.0)	56 (15.0)	1.00	—
Abortion History (Ref: No)					
	Yes	71 (69.6)	31 (30.4)	3.84(2.34–6.30)	< 0.001
	No	653 (89.8)	74 (10.2)	1.00	—
Family Infertility (Ref: No)					
	Yes	130 (73.0)	48 (27.0)	4.02(2.60–6.22)	< 0.001
	No	589 (91.6)	54 (8.4)	1.00	—
History of STI (Ref: No)					
	Yes	47 (92.2)	4 (7.8)	0.56(0.20–1.57)	0.268
	No	677 (87.0)	101 (13.0)	1.00	—
Cigarette Use (Ref: No)					
	Yes	12 (36.4)	21 (63.6)	15.75(7.05–35.19)	< 0.001
	No	712 (90.0)	79 (10.0)	1.00	—
Alcohol Use (Ref: No)					
	Yes	220 (89.8)	25 (10.2)	0.76(0.47–1.23)	0.267
	No	504 (87.0)	75 (13.0)	1.00	—
Khat Use (Ref: No)					
	Yes	69 (97.2)	2 (2.8)	0.19(0.05–0.79)	0.022
	No	652 (86.9)	98 (13.1)	1.00	—
Surgical History (Ref: No)					
	Yes	40 (80.0)	10 (20.0)	1.90(0.93–3.87)	0.078
	No	684 (88.4)	90 (11.6)	1.00	—

The proportion of infertility cases varied across districts, with higher proportions observed in Wachale (15.9%) and Girar Jarso (13.4%) compared to Dera (10.7%); however, these differences were not statistically significant.

Educational status showed a significant crude association with infertility. Women with primary education (COR = 1.83; 95% CI: 1.08–3.09) and diploma-level education or higher (COR = 1.89; 95% CI: 1.01–3.54) had higher odds of infertility compared to women with no formal education.

Women’s occupation was also associated with infertility, with merchants having higher odds compared to housewives (COR = 2.65; 95% CI: 1.59–4.41). Similarly, women from female-headed households had higher odds compared to those from male-headed households (COR = 2.37; 95% CI: 1.50–3.74).

Reproductive characteristics showed strong associations. Women with a history of abortion (COR = 3.84; 95% CI: 2.34–6.30) and those with a family history of infertility (COR = 4.02; 95% CI: 2.60–6.22) had higher odds of infertility.

Among behavioral factors, cigarette smoking showed a strong association (COR = 15.75; 95% CI: 7.05–35.19). Khat use was associated with lower odds (COR = 0.19; 95% CI: 0.05–0.79); however, this estimate is based on a small subgroup and should be interpreted cautiously.

Variables with *p* < 0.25 were considered candidates for multivariable analysis.

### Focus group discussion

Six focus group discussions (two per district), involving 36 participants, were conducted to complement the quantitative findings.

Participants consistently reported that infertility is highly stigmatized, with women often bearing disproportionate blame regardless of underlying causes. This aligns with the quantitative findings showing associations with socio-demographic factors such as occupation and household context. As one participant stated:

*“In our community*,* women are usually blamed when a couple cannot have children*,* even if the problem may come from the husband.”* (FGD participant, Wachale)

Irregular menstrual cycles, history of abortion, and multiple sexual partnerships were frequently cited as perceived contributors to infertility, consistent with observed quantitative associations. Behavioral factors such as cigarette smoking and alcohol use were also perceived as harmful to reproductive health. One participant noted:

*“Women who have irregular menstruation or a history of abortion are often believed to have difficulty conceiving.”* (FGD participant, Girar Jarso)

Limited access to reproductive health services, particularly in rural areas, was repeatedly emphasized. This contextual insight supports the inclusion of geographic variables (district and residence) in predictive models. A participant explained:

*“In rural areas*,* it is difficult to access health services related to infertility*,* and many women do not know where to seek help.”* (FGD participant, Dera)

Overall, the qualitative findings provide contextual insights that complement the quantitative associations, particularly regarding stigma, reproductive health perceptions, and access to care. These findings are exploratory and should be interpreted as supportive rather than confirmatory.

### Factors associated with infertility: firth penalized logistic regression

Table 3 presents the results of the Firth penalized logistic regression analysis of factors associated with infertility. After adjustment, district of residence remained significantly associated with infertility. Women in Girar Jarso (AOR = 2.90; 95% CI: 1.30–6.50) and Wachale (AOR = 2.70; 95% CI: 1.25–5.90) had higher odds of infertility compared to those in Dera. Age at first marriage showed a statistically significant inverse association, with each additional year associated with reduced odds of infertility (AOR = 0.92; 95% CI: 0.85–0.99).

**Table 3:** Firth penalized logistic regression analysis of factors associated with infertility

Variables	β	S.E.	Wald	Sign.	AOR	95% CI for AOR	
						Lower	Upper
Districts (Ref: Dera)							
G/Jarso	1.065	0.410	6.75	0.009	2.90	1.30	6.50
Wachale	0.993	0.398	6.23	0.013	2.70	1.25	5.90
Education Leve(Ref: No educat)							
Primary	-0.320	0.410	0.61	0.435	0.73	0.32	1.65
Secondary	-0.350	0.420	0.69	0.405	0.70	0.30	1.63
Diplo &above	-0.620	0.455	1.85	0.174	0.54	0.22	1.30
Age at first Marriage	-0.083	0.040	4.31	0.038	0.92	0.85	0.99
Menstruation cycle(Ref: irregular)							
Regular	-0.545	0.255	4.58	0.032	0.58	0.34	0.96
Abortion history (Ref: No)							
Yes	1.116	0.332	11.30	0.001	3.05	1.60	5.80
Family infertility(Ref: No)							
Yes	1.082	0.285	14.38	0.000	2.951	1.687	5.161
Number of Sexual Partners	1.116	0.332	11.30	0.001	3.05	1.60	5.80
Use cigarettes (Ref: No)							
Yes	1.917	0.470	16.65	0.000	6.80	2.60	17.50
HH head Sex (Ref: Male)							
Female	-0.150	0.335	0.20	0.655	0.86	0.45	1.68
Women occupation (Ref House wife)							
Farmer	0.500	0.420	1.42	0.233	1.65	0.72	3.80
Merchant	0.810	0.300	7.29	0.007	2.25	1.25	4.00
Government	0.330	0.450	0.54	0.462	1.39	0.58	3.35
Constant	-1.720	0.820	4.40	0.036	0.18		

Women with regular menstrual cycles had lower odds of infertility compared to those with irregular cycles (AOR = 0.58; 95% CI: 0.34–0.96). A history of abortion (AOR = 3.05; 95% CI: 1.60–5.80) and family history of infertility (AOR = 2.95; 95% CI: 1.68–5.160) were significantly associated with higher odds of infertility. Additionally, each additional sexual partner was associated with increased odds of infertility (AOR = 1.60; 95% CI: 1.15–2.20).

Cigarette smoking remained strongly associated with infertility (AOR = 6.80; 95% CI: 2.60–17.50), although the effect size was attenuated after applying Firth’s penalized regression, suggesting reduced small-sample bias compared to the standard logistic regression model.

Regarding occupation, women engaged in merchant activities had higher odds of infertility compared to housewives (AOR = 2.25; 95% CI: 1.25–4.00). Educational level, sex of household head, and other occupational categories were not statistically significant after adjustment.

Model diagnostics indicated no evidence of multicollinearity (all VIF < 10). The Hosmer–Lemeshow goodness-of-fit test was not statistically significant (*p* > 0.05), suggesting adequate model fit. The use of Firth penalized logistic regression helped to reduce small-sample bias and improve estimate stability; however, results should still be interpreted with caution due to limited event counts in some categories.

### Model performance comparison

Table 4 summarizes the predictive performance of logistic regression and machine learning models. All performance metrics were evaluated on an independent test dataset.

**Table 4:** Model comparison results

Model	Accuracy	Sensitivity	Specificity	AUC (95% CI)	AUC (CV Mean ± SD)	Brier Score	F1-score	Balanced Accuracy
Logistic Regression	0.738	0.771	0.50	0.691 (0.63–0.75)	0.71 ± 0.07	0.19	0.30	0.64
Random Forest	0.927	0.951	0.75	0.893 (0.85–0.93)	0.93 ± 0.04	0.12	0.52	0.85
XGBoost	0.915	0.938	0.75	0.888 (0.84–0.92)	0.88 ± 0.06	0.13	0.51	0.84
SVM	0.854	0.889	0.60	0.810 (0.76–0.86)	0.83 ± 0.07	0.16	0.38	0.74

Random Forest demonstrated the highest predictive performance among the evaluated models, with accuracy (0.927), sensitivity (0.951), and AUC (0.893), followed closely by XGBoost (AUC = 0.888). The support vector machine showed moderate performance (AUC = 0.810), while logistic regression exhibited the lowest overall performance (AUC = 0.691).

Sensitivity was highest for Random Forest (0.951), indicating a strong ability to correctly identify infertility cases. However, specificity was lower across models, particularly for logistic regression (0.50), indicating a reduced ability to correctly classify non-infertile individuals. This pattern suggests a tendency to misclassify non-infertile individuals, which may be influenced by class imbalance and the use of resampling techniques.

Despite the high accuracy and AUC values observed for machine learning models, the F1-scores were moderate (0.52 for Random Forest and 0.51 for XGBoost), indicating limited precision in identifying positive cases. This further reflects the effect of class imbalance and highlights the importance of evaluating multiple performance metrics beyond accuracy alone.

Model performance during training was further assessed using 5-fold cross-validation. The mean ± standard deviation (SD) of AUC across folds were as follows: Random Forest (0.930 ± 0.040), XGBoost (0.878 ± 0.060), SVM (0.830 ± 0.070), and logistic regression (0.710 ± 0.070) (Table 4). The relatively small standard deviations indicate stable model performance across folds. However, these results reflect internal validation only.

In addition to discrimination metrics, model calibration was evaluated using the Brier score, where lower values indicate better agreement between predicted probabilities and observed outcomes. Random Forest (0.12) and XGBoost (0.13) demonstrated lower Brier scores compared to logistic regression (0.19), indicating relatively better overall predictive accuracy.

Nevertheless, given the class imbalance, relatively small number of positive cases, and the use of resampling techniques, the observed performance—particularly for machine learning models—may be optimistic. Furthermore, as calibration and discrimination were assessed using internal validation only, these findings require external validation in independent populations before generalization.

Given the class imbalance in the dataset, characterized by a relatively small number of infertility cases, the ROSE (Random Over-Sampling Examples) technique was applied to improve the detection of the minority class. While this approach enhances model sensitivity, it may also influence precision and the calibration of predicted probabilities by altering the original class distribution.

To assess the robustness of the findings, model performance was additionally evaluated on the original (non-resampled) dataset (results not shown). This analysis yielded lower sensitivity but slightly improved specificity, highlighting the trade-off between correctly identifying infertility cases and minimizing false-positive classifications.

The moderate F1-scores observed across the machine learning models further reflect this trade-off and underscore the challenges associated with imbalanced data. Therefore, all performance metrics should be interpreted with caution. Future studies should explore alternative approaches, such as the Synthetic Minority Over-sampling Technique (SMOTE), cost-sensitive learning, or ensemble methods, as well as external validation using independent datasets to improve generalizability and model reliability.

**Fig. 2:**
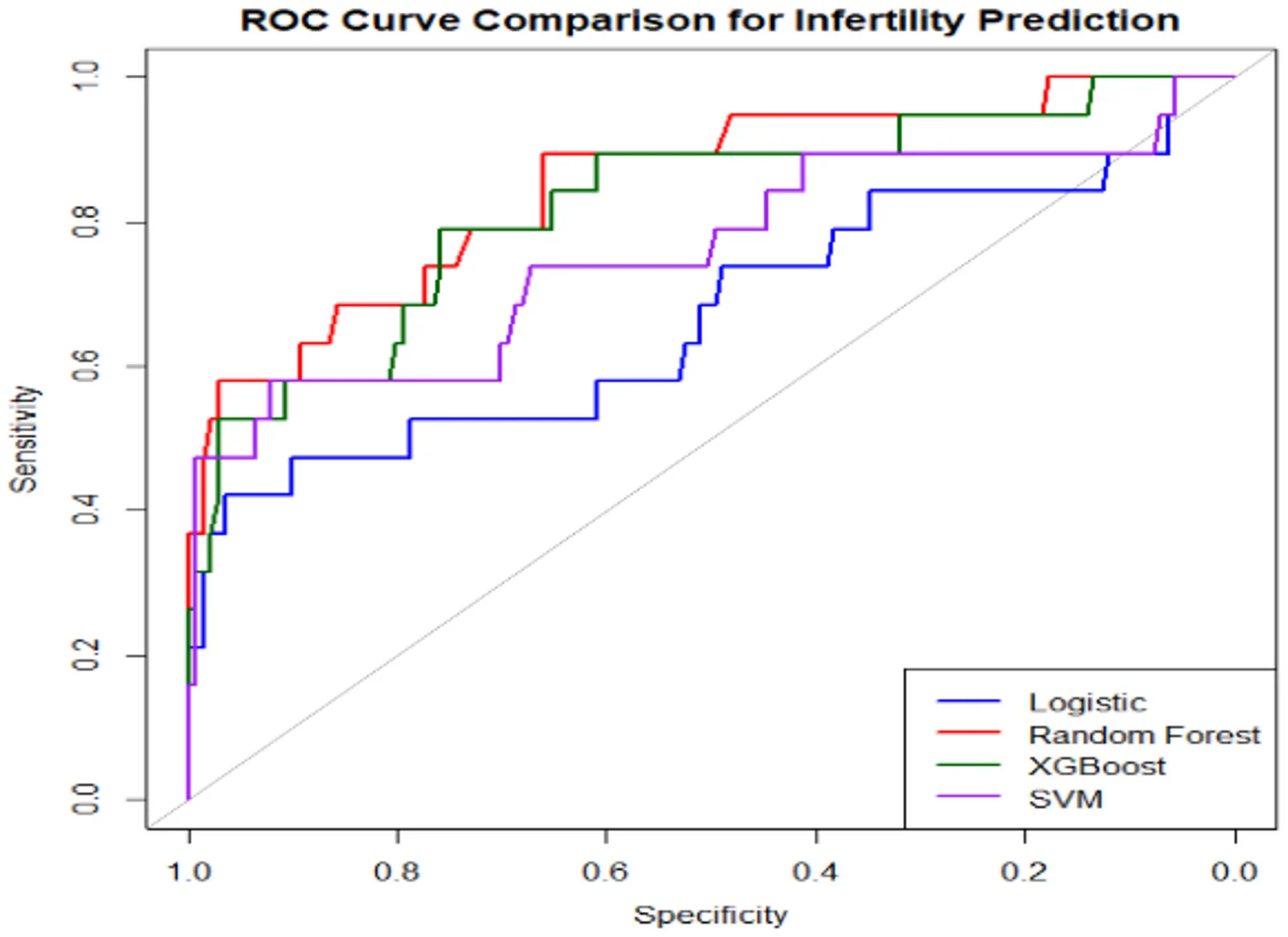
ROC curves for infertility prediction models

Figure 2 presents the ROC curves for all models, illustrating comparative discriminative performance based on the test dataset.

As shown in Fig. 2, the ROC curves for the four predictive models are presented to compare their discriminative performance. Random Forest exhibited the highest discriminative performance among the evaluated models; however, given the relatively small number of infertility cases and the use of internal validation only, this performance may be optimistic and should be interpreted with caution.

It should be noted that model performance was evaluated using internal validation only. Therefore, the reported predictive accuracy may not generalize to other populations or settings without external validation.

**Fig. 3:**
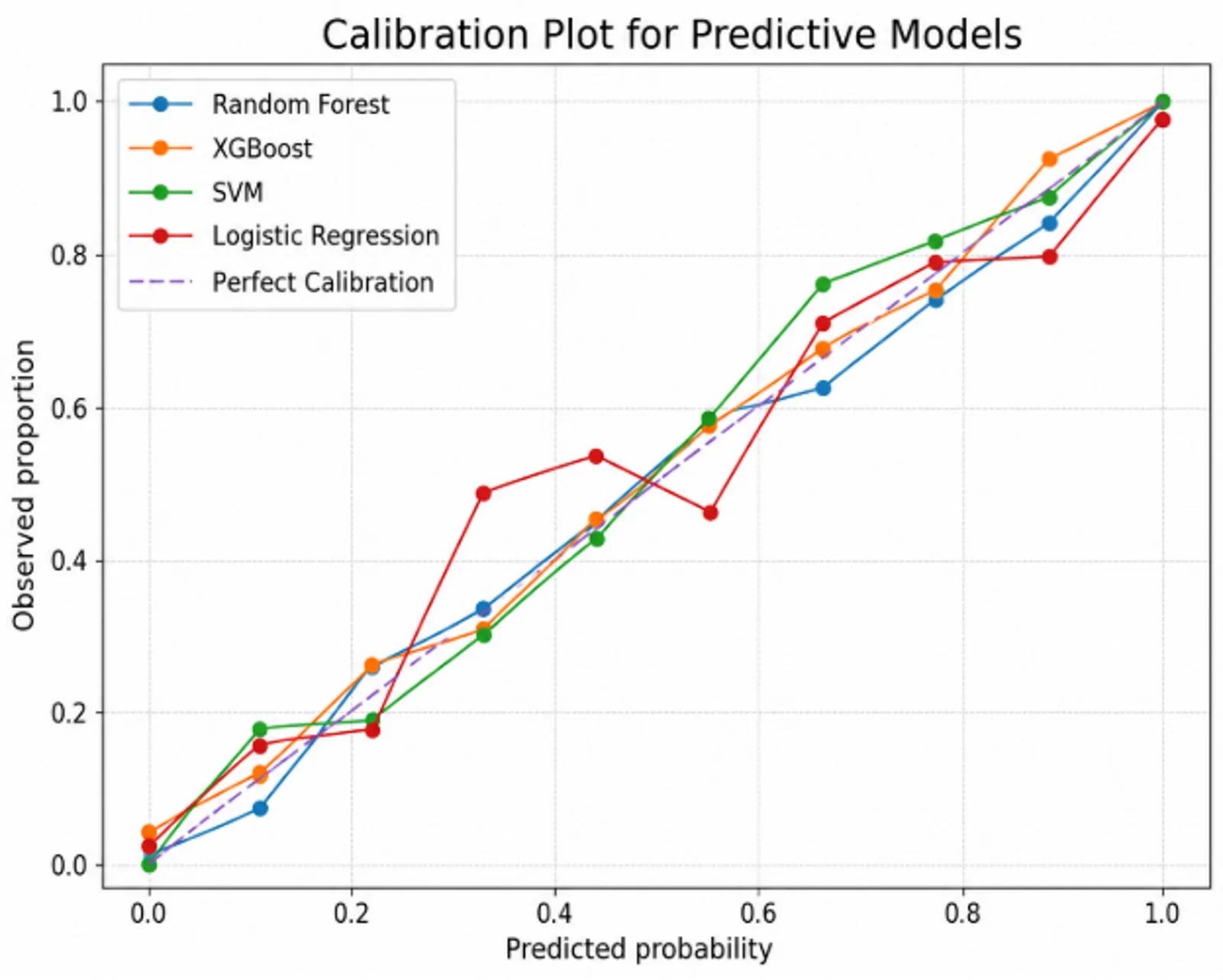
Calibration plot for predictive models showing the relationship between predicted probabilities and observed outcomes

The dashed diagonal line represents perfect calibration. Random Forest and XGBoost models demonstrated good calibration, with curves closely aligning with the ideal line, consistent with their lower Brier scores. The support vector machine showed moderate calibration, with slight deviations at higher probability ranges. In contrast, logistic regression exhibited greater deviation from the diagonal, particularly in the mid-probability range, indicating poorer calibration (Fig. 3). These findings suggest that machine learning models provide more reliable probability estimates compared to logistic regression; however, results should be interpreted with caution due to class imbalance and the use of internal validation only.

### Variable importance from the random forest model

**Fig. 4:**
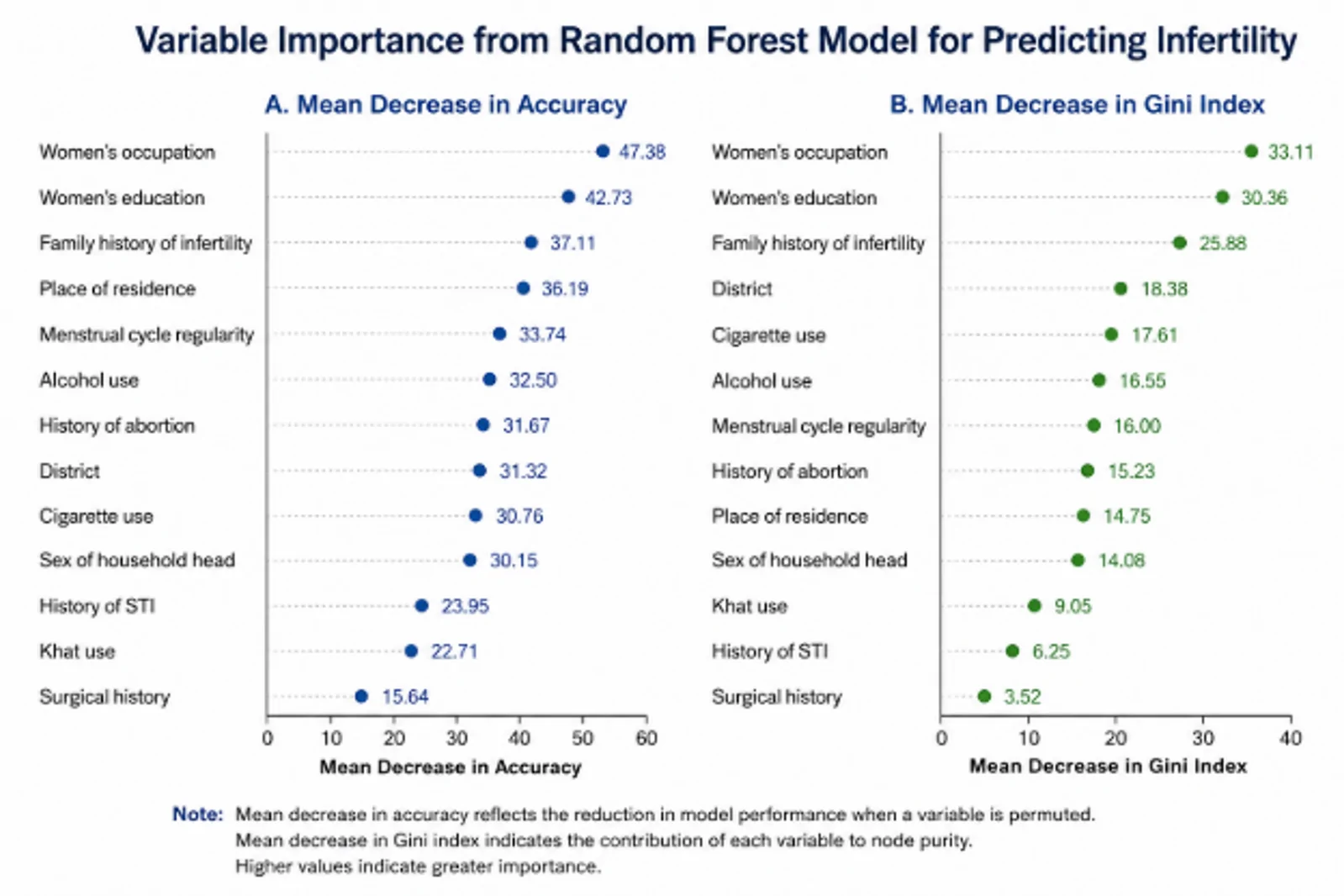
Variable importance from Random forest model

Figure 4 presents variable importance measures derived from the Random Forest model using mean decrease in accuracy and Gini index. These metrics reflect each variable’s contribution to prediction performance rather than causal or epidemiological importance.

Women’s occupation and educational level ranked among the most important predictors, followed by family history of infertility, place of residence, and menstrual cycle pattern. Behavioral factors such as cigarette and alcohol use also contributed to model performance. Variables such as history of sexually transmitted infections and surgical history showed relatively lower importance (Fig. 4).

Overall, these findings indicate that multiple socio-demographic, behavioral, and reproductive variables contribute to prediction performance. However, variable importance reflects predictive relevance within the model and may capture complex or non-linear relationships rather than causal effects.

## Discussions

This study assessed the prevalence of infertility and its associated factors among married women using a community-based cross-sectional design, complemented by machine learning models and qualitative findings. The overall prevalence of infertility was 12.7%, which is lower than estimates reported from facility-based studies in Addis Ababa (27.6%) and the Ethiopian Demographic and Health Survey (EDHS) 2016 (24.6%) [14], but comparable to estimates reported for low-income countries (12.6%) [1]. However, these comparisons should be interpreted cautiously due to differences in study design, definitions, and recall periods. The relatively lower prevalence observed in this study may reflect its community-based design, which captures a broader population, whereas facility-based studies may overrepresent women actively seeking infertility care.

The multivariable analysis identified district of residence, age at first marriage, menstrual cycle regularity, history of abortion, family history of infertility, number of sexual partners, cigarette smoking, and women’s occupation as factors associated with infertility. These findings were broadly consistent with patterns observed in the machine learning analysis. While logistic regression provides adjusted effect estimates suitable for epidemiological interpretation, machine learning models such as Random Forest identify variables that contribute most to prediction accuracy [26]. Importantly, variable importance in machine learning reflects predictive contribution rather than causal or independent epidemiological significance.

Variations in infertility across districts suggest the influence of geographic and contextual factors. Differences in access to reproductive health services, cultural practices, and environmental conditions may contribute to these patterns. The qualitative findings further highlighted disparities in access to reproductive healthcare, particularly in rural areas, supporting the relevance of contextual factors identified in the quantitative analysis [14, 31].

Age at first marriage showed an inverse association with infertility, indicating that women who married at younger ages were more likely to report infertility. This finding is consistent with previous studies [10, 15, 16] and may reflect biological immaturity, limited reproductive health knowledge, and constrained access to healthcare among women who marry early. However, given the cross-sectional design, the temporal direction of this relationship cannot be established.

Menstrual cycle regularity was also associated with infertility. Women reporting regular cycles were less likely to report infertility, consistent with existing evidence [3, 10, 16]. Irregular cycles may reflect underlying hormonal or ovulatory dysfunction, which are commonly linked to reduced fertility.

A history of abortion was associated with higher odds of infertility. This finding aligns with previous studies [6, 32, 33]; however, the cross-sectional design does not allow determination of whether abortion preceded infertility or vice versa. Similarly, a family history of infertility was strongly associated with infertility, suggesting potential roles of genetic susceptibility as well as shared environmental and behavioral factors [10].

The number of sexual partners was positively associated with infertility [3, 6], possibly due to increased exposure to sexually transmitted infections. Although self-reported STI history was not statistically significant in the adjusted model, underreporting and measurement limitations may have influenced this result.

Cigarette smoking was associated with higher odds of infertility; however, this estimate is based on a small subgroup and may be affected by sparse data bias. Nevertheless, the direction of association is consistent with existing literature linking tobacco use to impaired reproductive function [6, 8, 14]. Similarly, the observed inverse association between khat use and infertility is unlikely to represent a true protective effect and may instead reflect reporting bias, residual confounding, or reverse causality.

Women engaged in merchant activities had higher odds of infertility compared to housewives. This may reflect differences in occupational stress, work patterns, or environmental exposures, although the underlying mechanisms remain unclear and warrant further investigation [10, 31].

The qualitative findings highlighted the substantial social and psychological burden of infertility, including stigma, marital strain, and emotional distress. These findings complement the quantitative results by illustrating the broader social context in which infertility occurs, particularly in settings where childbearing is closely tied to women’s social status [4, 5, 7, 34].

Machine learning models demonstrated higher apparent discriminative performance within this dataset; however, these findings should be interpreted cautiously. The models were developed using internal validation only, and the relatively small number of infertility cases increases the risk of overfitting [26]. In addition, class imbalance and the use of resampling techniques may have influenced performance metrics. Calibration was assessed using the Brier score; however, because this was based on internal validation, the results may be optimistic and require external validation. Therefore, the reported performance may not generalize to other populations.

Overall, while logistic regression provides interpretable estimates for epidemiological analysis, machine learning models offer complementary insights by identifying patterns relevant for prediction. These approaches should be viewed as complementary rather than competing.

## Limitations of the study

This study has several limitations. First, the cross-sectional design limits the ability to establish temporal or causal relationships; therefore, the observed associations should be interpreted as correlational.

Second, infertility and related variables were based on self-reported data, which are subject to recall and social desirability biases, particularly for sensitive variables such as number of sexual partners, sexually transmitted infections, cigarette smoking, and khat use. This may have resulted in misclassification or underreporting. In addition, infertility was defined as self-reported failure to conceive over 12 months without clinical verification. Information on timing and frequency of intercourse, as well as male partner infertility, was not assessed, which may have contributed to outcome misclassification.

Third, the relatively small number of infertility cases (*n* = 105) may have reduced statistical power and produced unstable estimates, particularly for low-prevalence variables. This also increases the risk of sparse data bias and overfitting in machine learning models. Although class imbalance was addressed using the ROSE oversampling technique, such approaches do not introduce new information but instead resample existing data, which may amplify noise and contribute to optimistic model performance.

Fourth, important clinical and biological factors, including hormonal profiles, male partner fertility status, and detailed reproductive health history, were not included, which may result in residual confounding.

Fifth, although internal validation using k-fold cross-validation was performed, the models were not externally validated. Therefore, the reported predictive performance may be optimistic and may not generalize to other populations. Similarly, calibration assessment based on internal validation may overestimate model performance.

Finally, as the study was conducted in selected districts of North Shewa Zone, the generalizability of the findings may be limited. Moreover, machine learning models were used primarily for prediction, whereas logistic regression supports inference; thus, their findings should be interpreted as complementary rather than directly comparable.

Despite these limitations, this study provides valuable insights by integrating quantitative, machine learning, and qualitative approaches within a community-based framework.

## Conclusions and recommendations

### Conclusions

This study assessed the prevalence of infertility and its associated factors among married women in North Shewa Zone, Ethiopia, using a community-based design and an integrated analytical framework that combined conventional statistical methods with machine learning approaches. The prevalence of infertility was 12.7%, indicating that infertility remains an important public health concern with significant social, behavioral, and reproductive health implications.

Multivariable logistic regression identified district of residence, age at first marriage, menstrual cycle irregularity, history of abortion, family history of infertility, number of sexual partners, cigarette smoking, and women’s occupation as factors associated with infertility. These findings highlight the multifactorial nature of infertility, encompassing biological, behavioral, sociodemographic, and contextual determinants.

Machine learning models, particularly the Random Forest algorithm, demonstrated improved predictive performance compared with logistic regression. However, these results should be interpreted cautiously due to the absence of external validation and the relatively small number of infertility cases. Variable importance analysis identified key predictors, including menstrual cycle characteristics, family history of infertility, women’s occupation, and behavioral factors.

Overall, the consistency between regression and machine learning findings strengthens confidence in the identified determinants. The use of machine learning provided additional insights into complex relationships and interactions among variables. Integrating these approaches enhances both predictive performance and epidemiological understanding, supporting improved identification of high-risk groups and informing targeted public health interventions.

### Recommendations

Based on the findings of this study, the following recommendations are proposed:

#### Strengthening reproductive health services

Improve access to comprehensive reproductive and infertility-related services, particularly in rural and underserved areas. This includes strengthening primary healthcare systems, referral pathways, and availability of trained professionals for early detection and management.

#### Community awareness and stigma reduction

Implement community-based education programs to address misconceptions and stigma surrounding infertility. Engagement of community leaders, religious institutions, and both men and women is essential to promote shared responsibility for reproductive health.

#### Promote behavioral and lifestyle interventions

Target modifiable risk factors such as cigarette smoking and risky sexual behaviors through health education and prevention programs, including awareness of reproductive health consequences and promotion of safer practices.

#### Inform policy and program development

Integrate infertility prevention and management into existing reproductive and maternal health policies. Policies should address early marriage, reproductive health education, and equitable access to care.

#### Build healthcare capacity

Provide continuous training for healthcare professionals on infertility prevention, diagnosis, counseling, and referral to improve quality of care and reduce delays in treatment.

#### Encourage advanced analytical approaches

Researchers and health institutions should consider integrating machine learning methods with traditional statistical approaches to improve risk prediction and support evidence-based decision-making.

In conclusion, coordinated efforts involving government bodies, non-governmental organizations, health professionals, and community stakeholders are essential to reduce infertility-related stigma, strengthen preventive and curative reproductive health services, and improve fertility outcomes and overall well-being of women in Ethiopia.

## Data Availability

The data set used and/ or analyzed during the current study is available from the corresponding author on reasonable request.

## Abbreviations

AOR: Adjusted Odds Ratio
AUC: Area Under the Receiver Operating Characteristic Curve
CI: Confidence Interval
EDHS: Ethiopian Demographic and Health Survey
FGD: Focus Group Discussion
HH: Household
PPS: Probability Proportional to Size
RF: Random Forest
ROC: Receiver Operating Characteristic
ROSE: Random Over-Sampling Examples
SPSS: Statistical Package for the Social Sciences
STI: Sexually Transmitted Infection
SVM: Support Vector Machine
WHO: World Health Organization
XGBoost: Extreme Gradient Boosting
